# Supplementary material for: Online multiple hypothesis testing
Source: Stat Sci. Author manuscript; Available in PMC 2024 Jan 12. (PMC7615519; doi:10.1214/23-STS901)
Supplement: Appendix [file EMS185037-supplement-Appendix.pdf]

APPENDIX A: TEST LEVELS FOR SAFFRON

After choosing $w_0 < \alpha$, the test levels for SAFFRON with $\lambda_t \equiv \lambda$ being constant are defined as follows:

1. At each time t, define the number of candidates after the j-th rejection as $C_{j+} = C_{j+}(t) = \sum_{i=\tau_j+1}^{t-1} C_i$, where $C_t = \mathbb{1}\{P_t \le \lambda\}$.
2. SAFFRON starts with $\alpha_1 = \min\{(1-\lambda)\gamma_1 w_0, \lambda\}$. Subsequent levels are chosen as $\alpha_t = \min\{\lambda, \tilde{\alpha}_t\}$, where

$$\tilde{\alpha}_t = (1-\lambda)[w_0\gamma_{t-C_{0+}} + (\alpha - w_0)\gamma_{t-\tau_1-C_{1+}} + \alpha \sum_{j\ge 2} \gamma_{t-\tau_j-C_{j+}}].$$

Formulae for non-constant λ_t are in Ramdas et al. (2018).

APPENDIX B: TEST LEVELS FOR ADDIS

The testing levels for ADDIS are given by $\alpha_t = \min\{\lambda, \hat{\alpha}_t\}$, where

$$\hat{\alpha}_t = (\eta - \lambda)[w_0\gamma_{S^t-C_{0+}} + (\alpha - w_0)\gamma_{S^t-\tau_1^*-C_{1+}} + \alpha \sum_{j\ge 2} \gamma_{S^t-\tau_j^*-C_{j+}}]$$

and $S^t = \sum_{i<t} \mathbb{1}\{P_i \le \eta\}$, $\tau_j^* = \sum_{i\le\tau_j} \mathbb{1}\{P_i \le \eta\}$. See Tian and Ramdas (2019) for an alternative formulation of ADDIS where p-values greater than η are explicitly discarded, and the extension to a sequence $\{\eta_t\}_{t=1}^{\infty}$.

APPENDIX C: SIMULATION STUDY

Figure 6 shows the FDR of LORD++, SAFFRON, ADDIS and monotone AI compared with uncorrected testing, the BH procedure and alpha-spending, using the simulation set-up described in Section 3.2.
